# Efficacy and user acceptability of transfluthrin-treated sisal and hessian decorations for protecting against mosquito bites in outdoor bars

**DOI:** 10.1186/s13071-017-2132-6

**Published:** 2017-04-20

**Authors:** John P. Masalu, Marceline Finda, Fredros O. Okumu, Elihaika G. Minja, Arnold S. Mmbando, Maggy T. Sikulu-Lord, Sheila B. Ogoma

**Affiliations:** 1 Environmental Health and Ecological Sciences Department, Ifakara Health Institute, Off Mlabani Passage, Ifakara, P.O Box 53, Morogoro, United Republic of Tanzania; 2 School of Public Health, University of the Witwatersrand, Johannesburg, South Africa; 3 Institute of Biodiversity, Animal Health and Comparative Medicine, University of Glasgow, Glasgow, UK; 4 QIMR, Berghofer Medical Research Institute, Brisbane, Australia; 5 US National Research Council, National Academies of Sciences, Engineering and Medicine, Washington, DC USA

**Keywords:** Early-evening biting, Outdoor-biting, Residual malaria transmission, Vector control, Transfluthrin, Sisal decorative baskets and wall decorations

## Abstract

**Background:**

A number of mosquito vectors bite and rest outdoors, which contributes to sustained residual malaria transmission in endemic areas. Spatial repellents are thought to create a protective “bubble” within which mosquito bites are reduced and may be ideal for outdoor use. This study builds on previous studies that proved efficacy of transfluthrin-treated hessian strips against outdoor biting mosquitoes. The goal of this study was to modify strips into practical, attractive and acceptable transfluthrin treated sisal and hessian emanators that confer protection against potential infectious bites before people use bed nets especially in the early evening and outdoors. This study was conducted in Kilombero Valley, Ulanga District, south-eastern Tanzania.

**Results:**

The protective efficacy of hand-crafted transfluthrin-treated sisal decorative baskets and hessian wall decorations against early evening outdoor biting malaria vectors was measured by human landing catches (HLC) in outdoor bars during peak outdoor mosquito biting activity (19:00 to 23:00 h). Treated baskets and wall decorations reduced bites of *Anopheles arabiensis* mosquitoes by 89% (Relative Rate, RR = 0.11, 95% confidence interval, CI: 0.09–0.15, *P* < 0.001) and 86% (RR = 0.14, 95% CI: 0.11–0.18, *P* < 0.001), respectively. In addition, they significantly reduced exposure to outdoor bites of *Culex* spp. by 66% (RR = 0.34, 95% CI: 0.22–0.52, *P* < 0.001) and 56% (RR = 0.44, 95% CI: 0.29–0.66, *P* < 0.001), respectively.

**Conclusion:**

Locally hand-crafted transfluthrin-treated sisal decorative baskets and hessian wall decorations are readily acceptable and confer protection against outdoor biting malaria vectors in the early evening and outdoors: when people are resting on the verandas, porches or in outdoor social places such as bars and restaurants. Additional research can help support the use of such items as complementary interventions to expand protection to communities currently experiencing outdoor transmission of mosquito-borne pathogens.

## Background

Malaria elimination is undermined by residual transmission sustained by populations of mosquitoes that are less susceptible to insecticides used on long lasting insecticidal nets (LLINs) and indoor residual sprays (IRS) [1]. Progress towards elimination is further frustrated by increased outdoor biting and resting mosquitoes which preclude sufficient contact with lethal LLINs and IRS [2–4].

Novel, low-cost, scalable vector control tools that confer protection against early-evening indoor and outdoor-biting mosquitoes are urgently needed. Other than topical repellents [5, 6] and protective clothing [7], spatial repellents can be used in the early evening and throughout the night when LLINs are not in use. Apart from their toxicity, volatile airborne insecticides such as transfluthrin incapacitate mosquitoes and prevent them from locating hosts and obtaining blood meals [8] and are referred to as spatial repellents. Examples of spatial repellent delivery formats include pyrethroid-treated mosquito coils, vaporizer mats, aerosols, and paper strips as well as traditional practices such as burning and smoldering plants [9]. Efficacy of these tools is dependent on a number of factors including regular compliance by users and duration of efficacy. Efficacy of some repellent emanator products lasts a few days or weeks creating the need for frequent replacement or re-treatment of substrates with insecticides which may be impractical and costly in rural settings [10–12]. Affordable, long-lasting passive emanators that ideally create a mosquito free “bubble” that could protect a number of people found outdoors are required.

**Fig. 1:**
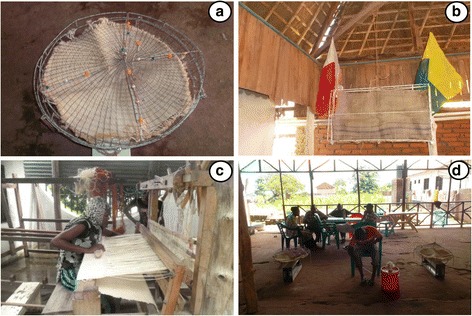
Sisal and hessian prototypes and the set up of HLC in bars. **a** A sisal decorative basket measuring approximately 0.6 m in diameter ~0.28 m^2^. **b** A hessian wall decoration flag approximately 0.7 m long and 0.4 m wide ~0.28 m^2^. **c** The process of weaving sisal strips. **d** Human landing catches conducted in a bar measuring approximately 11.73 m long, 6.30 m wide, and 4.71 m high at the roof apex

Recently, a new low-technology tool transfluthrin emanator comprising natural fiber hessian strips was shown to reduce human exposure to outdoor-biting malaria by more than 90% [10, 13, 14]. Although the previous format proved efficacious, the user was confined within 1 m^2^ area by a hessian strip (4.0 × 0.3 m) hang on wooden poles to create the square space [10] which was not practical for everyday use. The aim of the current study was to modify hessian strips [10, 13, 14] into practical, attractive, and readily acceptable formats (Fig. 1a, b) produced locally that can be used outdoors on verandahs, porches, outdoor bars, restaurants and during camping to confer protection against malaria vectors. We developed hand-crafted decorative baskets comprising of transfluthrin-treated sisal pieces enclosed in colorful Maasai beads and welded iron frames (Fig. 1a) 0.6 m in diameter and sisal wall decorations 0.4 × 0.7 m with flags of local Tanzanian football team (Fig. 1b) attached on the iron frame. Efficacy of these prototypes against outdoor mosquitoes was measured in bars using human landing catches (Fig. 1d), in Lupiro village in Ulanga District, south-eastern Tanzania. In addition, we assessed issues that influence user acceptability and uptake of transfluthrin-treated sisal baskets and wall decorations as vector control tools using focus group discussions.

## Methods

### Study area

The study was conducted in Lupiro village (8.385°S, 36.670°E), Ulanga District, south-eastern Tanzania [15] where annual rainfall is 1200–1600 mm with temperature ranging between 20.0 °C and 32.6 °C [15]. Experimental bars were located adjacent to rice irrigation farms characterized by high mosquito densities all year round. Previous bed net mass distribution campaigns within this region [16] have successfully reduced *Anopheles gambiae* (*sensu stricto*) population densities leaving *An. arabiensis* and *An. funestus* (*sensu lato*) mosquitoes as the main vectors [17–19]. A previous study indicated that *An. arabiensis* mosquitoes in this area were less susceptible to permethrin, lambda-cyhalothrin and deltamethrin [20].

### Local experimental bars

Experiments were conducted in 3 local outdoor bars during normal business daily working hours. Two of the bars had iron roofs while one was grass thatched. The walls were open including poles that supported the roofs. The bars were approximately 11.73 m long, 6.30 m wide, and 4.71 m high at the roof apex.

### Preparation of transfluthrin-treated fabrics and enclosing frames

Sisal and hessian fabrics 0.28 m^2^ each were treated with 5 ml technical grade 97% transfluthrin (Shenzhen Sunrising Industry Company, Shenzhen, China), detergent Axion® (Colgate-Palmolive (E.A) Ltd, Nairobi, Kenya) and water as previously described [10, 13, 14]. Control sisal and hessian pieces were soaked in a mixture of water and detergent only. All sisal formats were enclosed in welded iron frames baskets comprising of colorful Maasai beads (Fig. 1a) while hessian pieces were enclosed in a rectangular iron-welded metal frame along with untreated flags of local Tanzanian football teams attached on it (Fig. 1b). Iron frames were produced by Mcemuka Handcraft Group in Bagamoyo, Tanzania. The iron frames prevented direct skin contact with the transfluthrin-treated fabric.

### Efficacy of transfluthrin-treated sisal baskets and hessian wall decorations against mosquito bites in outdoor bars

**Fig. 2:**
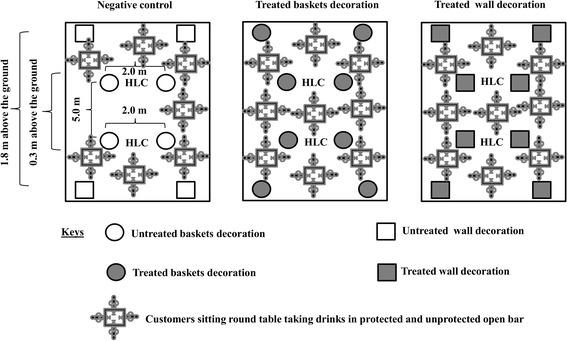
Schematic presentation of the experimental design. Two pairs of treatments were suspended 0.3 m and 1.8 m above the ground. Treatments (baskets or wall decorations) were placed equidistantly (2 m) on either side of the human conducting human landing catches. Two volunteers were allocated to each bar. They sat 5 m apart and exchanged positions at the top of every hour

Experiments were conducted between 26 May and 23 July 2015. In order to determine the relative protection of transfluthrin-treated baskets and wall decorations against mosquito bites in outdoor bars, treatments were randomly assigned to bars using a lottery method and thereafter, rotated between bars using a 3 × 3 Latin square design. Treatments included: (i) a control set up comprising untreated sisal baskets and wall decorations; (ii) 5 ml transfluthrin-treated sisal baskets; and (iii) 5 ml transfluthrin-treated hessian wall decorations. Three outdoor bars located approximately 400 m apart were selected in Lupiro village, Ulanga District, south-eastern Tanzania. The treatments were randomly allocated to the bars. Each bar contained 8 pieces of a single treatment (Fig. 2). Wall decorations and baskets were suspended approximately 0.3 m and 1.8 m above the ground (Fig. 2) to target mosquitoes that are attracted to human feet and to maximize contact with low wind speed so as to ensure maximum dispersion of the active ingredient within bars. After each experimental night (19:00–23:00 h), treatments were removed from the bars and stored in separate experimental huts with mean temperature 29.6 °C during the day and 26.4 °C at night, while mean relative humidity was 70.8% during the day and 81.8% at night to avoid cross-contamination between treatments. Experiments were not conducted on the fourth night to allow for wash-out of the active ingredients and to minimize carry-over effect of treatments between bars. A pair of trained male volunteers randomly allocated to each bar by choosing pieces of paper with bar identifier numbers conducted human landing catches from 19:00 to 23:00 h and changed positions (5.0 m apart within the bar premise) at the top of every hour (Fig. 1d). Therefore, a total of 6 volunteers collected mosquitoes simultaneously each night. In addition, volunteers counted the number of people present in each bar at the end of every mosquito collection hour. Volunteers moved between bars every night in pairs following a 3 × 3 Latin square design. Mosquitoes collected by each volunteer were morphologically identified to species level each morning: *An. gambiae* (*s.l.)* group and *An. funestus* (*s.l*.) or genus: *Culex* spp. and *Mansonia* spp. A sub-sample of *An. gambiae* (*s.l*.) and *An. funestus* (*s.l*.) was randomly selected and stored in micro-centrifuge tubes containing silica gel for further species identification by polymerize chain reaction (PCR) [21, 22]. Experiments were repeated five times over a total of 45 experimental nights, each time with freshly treated items.

### Acceptability of transfluthrin treated sisal and hessian decorative items by bar owners and regular customers through focus group discussions

Focus group discussions (FGDs) were used to evaluate two themes: (i) knowledge of participants regarding the burden of mosquito bites and malaria prevention; and (ii) perception towards effectiveness of transfluthrin-treated sisal decorations in reducing mosquito bites and malaria transmission. A total of 3 FGDs were held in Lupiro village with bar workers and regular customers who visited experimental bars between 26 May and 23 July 2015. Purposive sampling was used to select focus group participants. All participants were adults and permanent residents of Lupiro village. They included frequent customers or employees at selected local experimental bars. The discussions were semi-structured; the facilitator asked a question and the participants discussed knowledge, views and comments. The discussions were facilitated by two social scientists; a note taker and an assistant. The purpose of the discussions was explained and verbal and written informed consents were obtained from the participants. Discussions were all conducted in the local language (Swahili) and lasted between 40 and 60 min each.

### Data analysis

Reduced outdoor biting rate was determined by comparing mean mosquito catches by HLC volunteers in bars with transfluthrin-treated items *versus* those bars that had untreated items. The number of mosquitoes was fitted to a generalized linear mixed effects models (GLMMs) with Poisson distributions in R statistical software version 3.1.3, with *lme4* package [23]. A random effect was fitted for each row of data in order to account for the over-dispersed nature of experimental mosquito count data. Random effects included: date of experiment, volunteer identification code, identity of the bar and number of people in the bar. The treatment (control, treated baskets and wall decorations) was treated as a fixed factor.

Audio tapes were used to record all focus group discussions. A trained anthropologist transcribed verbatim and translated the recordings into English for further analysis. Detailed reports of all the discussions were organized according to emerging themes and trends were summarized. Quotes from participants are presented.

## Results

### Efficacy of transfluthrin-treated sisal baskets and hessian wall decorations against mosquito bites in outdoor bars

#### Identification of mosquito species

The total number of mosquitoes collected in the bars was 6214. They included 5302 *An. arabiensis,* 58 *An. funestus* (*s.l*.), 7 *An. pharoensis*, 8 *An. ziemann*, 828 *Culex* spp., 10 *Mansonia* spp., and 1 *Coquilettidia* spp. Polymerize chain reaction (PCR) for species identification was conducted on 249 *An. gambiae* (*s.l*.), with 78.3% (195/249) successful amplifications, that were all identified as *An. arabiensis*. For *An. funestus* group, 21 samples were analyzed, with 85.7% (18/21) successful amplifications, of which 77.8% (14/18) were *An. funestus* (*sensu stricto*) and 22.2% (4/18) were *An. rivulorum.*

#### Reduction of mosquito biting rates within outdoor bars

**Fig. 3:**
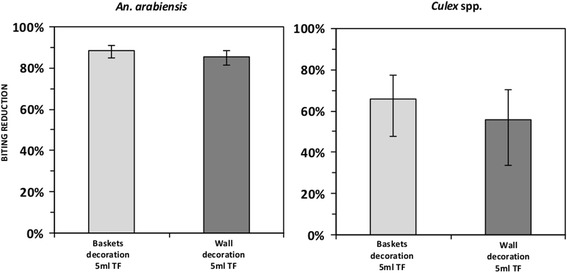
Relative protection of transfluthrin-treated sisal baskets and wall decorations against outdoor bites of *An. arabiensis* and *Culex* spp. The errors bars represent the 95% confidence intervals. *Abbreviation*: TF, transfluthrin

As detailed in Table 1 and Fig. 3, both 5 ml transfluthrin-treated sisal baskets and hessian wall decorations reduced bites of *An. arabiensis* mosquitoes by 89% (Relative rate, RR = 0.11, 95% confidence interval, CI: 0.09–0.15, *P* < 0.001) and 86% (RR = 0.14, 95% CI: 0.11–0.18, *P* < 0.001), respectively. In addition, treated baskets and wall decorations reduced exposure to *Culex* spp. bites by 66% (RR = 0.34, 95% CI: 0.22–0.52, *P* < 0.001) and 56% (RR = 0.44, 95% CI: 0.29–0.66, *P* < 0.001), respectively. The mean number of people protected per bar per night was 49.

**Table 1:** Mean collection of mosquitoes per person per night between bars that had transfluthrin treated and untreated sisal decorative items

Treatment	*N*	Mean number (adjusted)	95% CI	*Z*-value	*P*-value
*An. arabiensis*					
Untreated bd and wd	4,079	70.16	37.91–130.16	13.48	< 0.001
5 ml TF bd	583	8.03	4.29–15.04	6.51	< 0.001
5 ml TF wd	640	9.98	5.34–18.65	7.22	< 0.001
*Culex* spp.					
Untreated bd and wd	370	5.06	3.19–8.01	6.91	< 0.001
5 ml TF bd	233	1.71	1.02–2.86	2.05	0.040
5 ml TF wd	225	2.23	1.37–3.64	3.21	< 0.001

*Abbreviations*: *N* total number of mosquitoes collected, *CI* confidence interval, *bd* basket decoration, *wd* wall decoration, *TF* transfluthrin

### Acceptability of transfluthrin treated sisal decorative items by restaurant/bar owners and regular customers

The aim of the FGD was to assess the participants’ knowledge of malaria burden, its vector and their views and perceptions regarding efficacy of transfluthrin-treated sisal/hessian decorations. Each of FGD session had 8 participants ranging in age between 20 and 60 years of age. Seventy-nine percent (19) of the participants were male while 21% (5) were female. Several themes arose during the discussions as explained below.

#### Children are at a higher risk of suffering from malaria

Participants seemed to be fairly informed about who bore the heaviest burden of the disease. They stated that children were at the highest risk: *“The proportion of children taken to the hospital is so much higher than that of adults going for malaria treatment. This disease is especially harder on children than adults.”* The reasons given varied from low immunity, small size, difficulty in warding off mosquitoes and lack of knowledge of symptoms of malaria.

#### Poverty largely contributes to persisting malaria transmission

Participants were in agreement that poverty played a big role in maintaining high levels of malaria transmission: *“Even now if you go around you will find that there are a lot of people with no mosquito nets because they cannot afford them. This leaves even the children unprotected. Children do not have to be in bars at night to be at risk of malaria transmission.”* Lack of knowledge on prevention methods was also mentioned as another barrier.

#### Malaria is spread by a female mosquito

Most of the participants were in agreement that malaria was transmitted by female *Anopheles* mosquitoes, and that in order for the mosquito to infect a healthy person, it had to have bitten a person infected with: *“If someone has malaria, and mosquitoes bite them, they then carry malaria parasite from that person. These mosquitoes can pass on those parasites to other people through bites. That is how I understand malaria spreads.”* They mentioned that mosquitoes preferred breeding in places with water, where people fetch water and in little ponds and bushes.

#### Bed nets are the major protection methods

Participants mentioned that bed nets were the main method used to protect against mosquito bites: *“At home I make sure that I sleep under the [mosquito] net.” “At home I make sure that my family uses nets every night, even when it is too hot. That is what most other people use too. We are given nets for free sometimes, and sometimes the price is reduced, then almost everyone has a net.”* Other personal protection methods mentioned included: long-sleeved clothing and topical repellents: *“When I go out at night sometimes I use the Chinese topical mosquito repellents. But I don’t know how good it is, or if it has other effects. Other times I just wear long sleeve clothing.”* Keeping the environment clean and cutting grass to reduce mosquito breeding and resting sites was also mentioned.

#### Non-peridomestic nighttime activities put people at an increased risk of getting malaria

Participants mentioned that people who were more likely to be outside at night were at the highest risk. They mentioned farmers (during the farming seasons), night guards, night sex workers and people living close to mosquito breeding sites. The participants mentioned that some events such as funerals, parties and other gatherings increased exposure to mosquito bites and malaria transmission. Clothing worn by women also increased their exposure to bites. Some comments from participants include: *“What I understand is that mosquitoes like any place with a gathering of people. Mosquitoes are not as dumb as we think; they know where they can get food, so they follow people.” “Mosquitoes like where there are a lot of people; they know that they can get blood from at least one person.”*

#### Perception towards effectiveness of transfluthrin-treated sisal decorations in reducing mosquito bites and malaria transmission

All participants had positive comments regarding transfluthrin-treated sisal baskets and hessian wall decorations. They expressed that since the introduction of the transfluthrin-treated sisal baskets and hessian wall decorations; they could sit and work in peace, with no disturbance from mosquitoes. The customers stated that they enjoyed their evenings and nights while bar workers remarked that they could work all night without being bothered by mosquitoes. Anecdotes describing participants’ observations of changing mosquito biting pressure with transfluthrin treated and control prototypes. *“I do not know how it works, but that is okay, because there are no mosquitoes. Before having this trap I would go home with very itchy feet from mosquito bites. In the morning I would wake up with many bumps from mosquito bites. But now I can sit peacefully and enjoy my drink.” “Since they put it there - at my bar, I am no longer bothered by mosquitoes. My customers are grateful too, that they can enjoy their drinks.”*

They indicated that decorations would be more effective in places with gatherings of people, mostly in places where people were likely to be found sitting in one place for long periods of time. Some of the places mentioned were churches, mosques, restaurants, markets, bars, night clubs and during parties or funerals: *“A trap like these would be greatly useful in weddings, or in funerals. In places like these people are either very sad or very happy, many are not conscious of mosquitoes around them. It would be great to have something like this that protects people throughout.”*

Participants had different views regarding the mode of action of transfluthrin-treated baskets and wall decorations. Some thought they attracted and killed mosquitoes while others thought they repelled mosquitoes, and some did not know how they worked. Some comments from some of the participants include: *“This thing confuses me. I sit close to it many times to see if it actually works, and I think it does because mosquitoes do not bother me. But what I do not understand and I have even asked the people who come, is that, if you bring this trap to chase away mosquitoes, then why are you sitting here still trying to catch the mosquitoes? I do not understand this.” “It chases mosquitoes away. It has mosquito repellent in it.” “I do not think this thing repels mosquitoes. I see they put it there at the bar, and then there are people who come with buckets to collect mosquitoes. I watch them, and every day I see they catch mosquitoes. So then I know that this thing does not repel mosquitoes because if it did, then how come those people still catch mosquitoes?”*

Overall all the people interviewed expressed that they would like to own one of the decorations for use at homes, especially in the evening hours when most of the household members were outdoors for various reasons. Some of the responses included: *“I would very much like to have this in my house. It looks good in the living room, and it protects you. I would like to own it.” “I cannot think of anyone who would not want to have one of these decorations. There is not a single person here who will say that he has never had malaria, or who sees not know what it feels like to have malaria, or have a child sick of malaria. I would be very happy to have something like this to protect me and my family, especially on days when it is too hot to use a net.”*

#### Improvement of the sisal decorations: Make it attractive and safe

When asked about how to improve the treated decorations, many respondents said that the decorations were good as they were because they seemed to be efficacious. However, a few respondents suggested improving the outlook of the decorations: *“As my friends have said, these traps are best in places where people gather, like in bars or churches. In places like these people like to be in appealing environment, so it would be great if you could make them look good so that people can look and admire them.”*

Others preferred fabric made decorations rather than wire frames that could possibly increase the risk of accidents: *“These traps are placed in areas where people meet and drink. Anything can happen in these environments; people may get into fight and use these as weapons, or someone may fall in it and get scratched. So if possible it would be great if you could make them with materials that are not wires, like wood, plastic or other things.”*

## Discussion

The present study demonstrated that transfluthrin-treated decorative baskets and wall decorations reduced exposure to bites of *An. arabiensis* and *Culex* spp. mosquitoes by more than 80 and 60%, respectively (Fig. 3). Moreover, both treatments demonstrated reduction in mean biting rates for about nine folds and three folds for *An. arabiensis* and *Culex* spp. mosquitoes respectively (Table 1). This study highlights the potential of hand-crafted transfluthrin-treated sisal/hessian baskets and wall decorations for use against potential malaria vectors and nuisance bites in bars and similar outdoor settings such as verandas, porches and during outdoor camping. Other studies reported efficacy of metofluthrin-treated emanators against outdoor-biting *Culex* spp. mosquitoes in Indonesia lasting 11–15 weeks [24]. Short-term efficacy of emanators requires frequent re-treatment of substrates with the insecticides or replacement which may be costly for people living in low-middle income countries. Luckily, the long-term protective efficacy of treated hessian fabric spanning over a year has been proven [14] implying that these prototypes may be affordable. Nevertheless, further studies that analyze the cost-benefit of the production and use of these prototypes in different communities should be prioritized.

Efficacy of repellent products depends on acceptance, uptake and compliance by users [11, 12, 25]. The current study aimed to improve the initial prototype [10, 13, 14] into attractive, readily acceptable practical formats by crafting attractive colorful decorations that also reduce mosquito bites. It was evident from the FGDs that participants found the new prototypes acceptable, appealing and efficacious in terms of protection from mosquito bites. Interestingly, they clearly noted the reduction in mosquitoes biting pressure in bars that had treated and untreated decorations. The perceived difference is attributed to rotation of treated and untreated prototypes between bars. However, the mechanism by which the prototypes reduced bites was not well understood. Some of the participants thought they either repelled or attracted mosquitoes that were then trapped by HLC. This confusion is likely to have been brought about by the fact that we conducted HLC in bars during efficacy evaluation. Despite lack of the scientific knowledge on the mode of action of the sisal/hessian decorations, participants were in agreement that these items reduced exposure to bites. However, suggestions to improve their appearance and safety were made.

Approximately 49 customers were in selected study bars each night during experiments. This implied that the treated baskets and wall decorations conferred area wide protection to customers as was confirmed by reduction of mosquitoes captured by HLC conducted in bars and also the perceived reduction of bites as confirmed by FGDs. This is consistent with the previous study which indicated that transfluthrin-treated hessian strips reduced exposure to *An. arabiensis* mosquitoes up to a radius of 5 m [14]. This indicated that all customers and bar attendants within the bars were protected irrespective of their position.

The sisal decorative baskets and wall decorations used here were produced by local community groups in Tanzania (Fig. 1c). Several communities in East Africa are already earning a living through production of sisal-based household items such as sleeping mats, carpets, door mats and wall decorations. Mass production of these products for use against mosquito bites is likely to provide a potential business opportunity for local communities and should be investigated. In addition, further studies to evaluate the cost of producing these prototypes locally or even at industrial level should be conducted.

## Conclusions

Transfluthrin-treated sisal or hessian decorations could be used to confer protection against outdoor-biting malaria vectors, effectively complementing existing interventions like LLINs and IRS, especially where residual malaria transmission occurs predominantly outdoors. Further studies should be conducted to measure the epidemiological impact of these prototypes as well as determine the cost of production. These tools have potential benefits which include: (i) release of the active ingredient at ambient tropical temperatures therefore do not require external electrical heating or combustion; (ii) protecting multiple users occupying a particular space; and (iii) being readily acceptable by users [25].

## Abbreviations

CI: Confidence interval
FGDs: Focus group discussions
GLMMs: Generalized linear mixed effects models
HLC: Human landing catch
IHI: Ifakara Health Institute
IRS: Indoor residual spraying
LLINs: Long lasting insecticidal nets
mRDT: Malaria rapid diagnostics test
NIMR: National Institute for Medical Research
RR: Relative rate
